# Key strategies for determining surgical resection margins in OSCC

**DOI:** 10.1097/JS9.0000000000002704

**Published:** 2025-06-20

**Authors:** Rui Shi, Wen-Hao Ren, Ke-Qian Zhi

**Affiliations:** a Department of Oral and Maxillofacial Reconstruction, the Affiliated Hospital of Qingdao University, Qingdao, China; b School of Stomatology of Qingdao University, Qingdao, China; c Department of Oral and Maxillofacial Surgery, the Affiliated Hospital of Qingdao University, Qingdao, China; d Key Lab of Oral Clinical Medicine, The Affiliated Hospital of Qingdao University, Qingdao, Shandong, China

*Dear Editor,*

We commend the authors for their methodical analysis in the recent review article, *“Surgical Margins in Head and Neck Squamous Cell Carcinoma: A Narrative Review,”* which provides a critical evaluation of the evidentiary thresholds and multifactorial determinants influencing surgical margin (SM) adequacy in head and neck squamous cell carcinoma (HNSCC). By synthesizing evidence from heterogeneous clinical contexts—including anatomical constraints, histopathological variability, and intraoperative decision-making paradigms—this work not only clarifies oncologically safe resection boundaries but also offers practical guidance for optimizing therapeutic outcomes in oral squamous cell carcinoma (OSCC). Building upon this foundation, we aim to supplement these findings with clinically relevant observations derived from our institutional experience in managing advanced buccal and gingivobuccal OSCC cases^[1]^.

The treatment of buccal oral squamous cell carcinoma (OSCC) necessitates a delicate equilibrium between oncologic radicality and the preservation of both functional anatomy and aesthetic contours^[2]^. For anterior buccal lesions, a minimum 1-cm three-dimensional safety margin must be rigorously maintained, with particular emphasis on microsurgical precision when tumors encroach upon critical structures such as the oral commissure. In such cases, wedge resection under high-magnification guidance enables selective preservation of the orbicularis oris muscle fibers and labial vascular arborization, thereby mitigating risks of postoperative microstomia or disfiguring scar formation. Posterior buccal tumors demonstrating infiltration into the pterygomandibular space or infratemporal fossa mandate a transcranial approach. Here, retrograde dissection—initiating with detachment of the medial and lateral pterygoid muscles at their zygomatic root origins—prevents iatrogenic retraction of tumor-laden muscle stumps into the skull base, a phenomenon implicated in locoregional recurrence. Intraoperative adjuncts, including frozen-section margin analysis and temporary mandibular ramus osteotomy, optimize tumor visualization. Defect reconstruction should be tailored to anatomical complexity, with radial forearm free flaps preferred for moderate-sized defects and anterolateral thigh flaps reserved for extensive composite losses.

Gingival carcinomas breaching the mandibular medullary cavity require definitive management via segmental mandibulectomy, a strategy demonstrably superior to marginal (“box”) osteotomy in both oncologic and functional outcomes^[3]^. While box osteotomy preserves the cortical envelope, its inherent biomechanical frailty—stemming from cancellous bone removal (50–70% load-bearing capacity reduction) and cortical devascularization—predisposes to pathologic fractures (25–30% incidence at 3-year follow-up). Furthermore, the residual cortical “shell” lacks sufficient trabecular volume for osseointegrated implants and undergoes accelerated resorption due to stress-shielding effects. In contrast, segmental resection with ≥1-cm bony margins ensures complete tumor extirpation while maintaining mandibular continuity. Reconstruction via bridging titanium plates or vascularized fibular flaps restores biomechanical integrity and provides adequate bone stock for secondary implant rehabilitation (vertical height ≥10 mm; width ≥6 mm). Advanced cases involving the inferior alveolar canal necessitate adjunctive condylar-subvertical osteotomy to secure tumor-free neural margins.

Despite inducing radiographic tumor regression, neoadjuvant chemoradiation does not license reduction in surgical resection extent. Histopathologic studies reveal persistent viable tumor cells or lymphovascular emboli at margins in 30–40% of cases, even when imaging suggests complete response^[4]^. Consequently, resection boundaries must be determined by the original tumor dimensions, with en bloc excision adhering to 1.5-cm three-dimensional margins. Neck dissection should comprehensively address tumor-draining nodal basins (Levels I–V for advanced lesions). Intraoperative frozen-section mapping is indispensable in post-neoadjuvant fibrotic beds to detect discontinuous “skip lesions.” Reconstruction of irradiated defects demands vascularized tissue transfer (e.g., anterolateral thigh or latissimus dorsi flaps) to counteract impaired wound healing and minimize fistula formation. Adherence to these protocols, as endorsed by NCCN guidelines, correlates with significant reductions in local recurrence (28% vs. 12%) and distant metastasis (18% vs. 9%)^[5]^.

This review synthesizes evidence-based protocols for OSCC management, emphasizing the integration of radical resection principles with functional rehabilitation strategies. Key advances include anatomically tailored buccal resection algorithms, biomechanically optimized mandibular reconstruction, and neoadjuvant-to-surgical contingency planning. Crucially, the dogma of “no margin reduction post-neoadjuvant therapy” remains sacrosanct, underscoring the irreplaceable role of histopathologic verification in oncologic surgery.

## Data Availability

Not applicable.
